# Cognitive-Behavioral Styles of Dementia Care Management: Targeting and Tailoring to Style

**DOI:** 10.1093/geroni/igab046.206

**Published:** 2021-12-17

**Authors:** Amanda Leggett, Hyungjung Koo, Cathleen Connell, Laura Gitlin, Helen Kale

**Affiliations:** 1 University of Michigan, Ypsilanti, Michigan, United States; 2 University of Michigan, Ann Arbor, Michigan, United States; 3 University of Michigan School of Public Health, Ann Arbor, Michigan, United States; 4 Drexel University, College of Nursing and Health Professions, Drexel University, Pennsylvania, United States; 5 UC Davis, Sacramento, California, United States

## Abstract

Despite an extensive literature on the stress process of caregiving, little attention has focused on how caregivers provide care (caregiving styles). To explore caregiving styles among 100 primary caregivers for persons living with dementia, we utilize k-modes machine learning analysis. This technique clusters caregiver’s use of behavioral (Dementia Management Strategies Scale; criticism, active management, encouragement) and cognitive (Caregiver Readiness Scale; understanding, adaptability) approaches into style profiles. Three styles were identified: Managers (n=25; high use of criticism, moderate use of active management and encouragement, poor understanding and adaptability), Adapters (n=48; low use of criticism, high use of adaptive management and encouragement, good understanding and adaptability), and Avoiders (n=27; low use of all behavioral strategies, moderate adaptability and understanding). Styles differ by demographic and care characteristics. Findings suggest that caregivers have variable approaches to care that are measurable, thus, targeting/tailoring interventions to caregiver style could be an effective approach.

